# 5,5′-[1,4-Phenyl­enebis(methyl­enesulfanedi­yl)]bis­[1,3,4-thia­diazol-2(3*H*)-one] dimethyl sulfoxide disolvate

**DOI:** 10.1107/S1600536812006289

**Published:** 2012-02-17

**Authors:** Sung Kwon Kang, Nam Sook Cho, Siyoung Jang

**Affiliations:** a Department of Chemistry, Chungnam National University, Daejeon 305-764, Republic of Korea

## Abstract

The asymmetric unit of the title compound, C_12_H_10_N_4_O_2_S_4_·2C_2_H_6_OS, contains one half of the *p*-xylene mol­ecule and one dimethyl sulfoxide mol­ecule. The *p*-xylene mol­ecule is located about a crystallographic inversion centre. In the mol­ecule, the thia­diazole and benzene rings are almost perpendicular to one another, with a dihedral angle of 88.95 (6)°. In the crystal, an N—H⋯O hydrogen bond is observed between the two components. The dimethyl sulfoxide mol­ecule is disordered over two orientations with an occupancy ratio of 0.879 (1):0.121 (1).

## Related literature

For general background to polydentate macrocyclic compounds, see: Dietrich *et al.* (1993 ▶); Vogle (1991 ▶). For the synthesis and reactivity of thia­diazole derivatives, see: Cho *et al.* (1998 ▶, 1999 ▶, 2001 ▶).
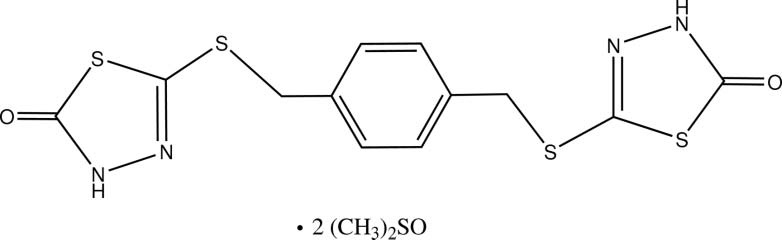


## Experimental

### 

#### Crystal data

C_12_H_10_N_4_O_2_S_4_·2C_2_H_6_OS
*M* *_r_* = 526.74
Triclinic, 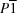

*a* = 7.5723 (15) Å
*b* = 8.3258 (17) Å
*c* = 10.346 (2) Å
α = 109.70 (4)°
β = 95.74 (3)°
γ = 91.15 (3)°
*V* = 610.0 (2) Å^3^
*Z* = 1
Mo *K*α radiation
μ = 0.59 mm^−1^
*T* = 296 K
0.18 × 0.17 × 0.12 mm

#### Data collection

Bruker APEXII CCD diffractometer
Absorption correction: multi-scan (*SADABS*; Bruker, 2002 ▶) *T*_min_ = 0.895, *T*_max_ = 0.923
21342 measured reflections
3039 independent reflections
2124 reflections with *I* > 2σ(*I*)
*R*_int_ = 0.083

#### Refinement

*R*[*F* ^2^ > 2σ(*F* ^2^)] = 0.034
*wR*(*F* ^2^) = 0.100
*S* = 1.02
3039 reflections
162 parameters
3 restraints
H atoms treated by a mixture of independent and constrained refinement
Δρ_max_ = 0.16 e Å^−3^
Δρ_min_ = −0.22 e Å^−3^

### 

Data collection: *SMART* (Bruker, 2002 ▶); cell refinement: *SAINT* (Bruker, 2002 ▶); data reduction: *SAINT*; program(s) used to solve structure: *SHELXS97* (Sheldrick, 2008 ▶); program(s) used to refine structure: *SHELXL97* (Sheldrick, 2008 ▶); molecular graphics: *ORTEP-3 for Windows* (Farrugia, 1997 ▶); software used to prepare material for publication: *WinGX* (Farrugia, 1999 ▶).

## Supplementary Material

Crystal structure: contains datablock(s) global, I. DOI: 10.1107/S1600536812006289/is5071sup1.cif

Structure factors: contains datablock(s) I. DOI: 10.1107/S1600536812006289/is5071Isup2.hkl

Supplementary material file. DOI: 10.1107/S1600536812006289/is5071Isup3.cml

Additional supplementary materials:  crystallographic information; 3D view; checkCIF report

## Figures and Tables

**Table 1:** Hydrogen-bond geometry (Å, °)

*D*—H⋯*A*	*D*—H	H⋯*A*	*D*⋯*A*	*D*—H⋯*A*
N3—H3⋯O13	0.91 (2)	1.83 (2)	2.742 (3)	175.6 (19)

**Fig. 1.**
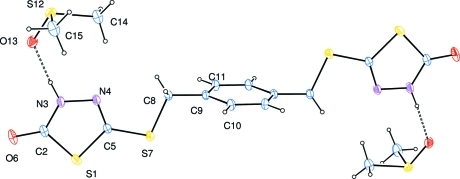
Molecular structure of the title compound, showing the atom-numbering scheme and 30% probability ellipsoids. Intermolecular N—H···O hydrogen bonds are indicated by dashed lines. Only major components of the disordered dimethyl sulfoxide molecule are shown.

**Fig. 2.**
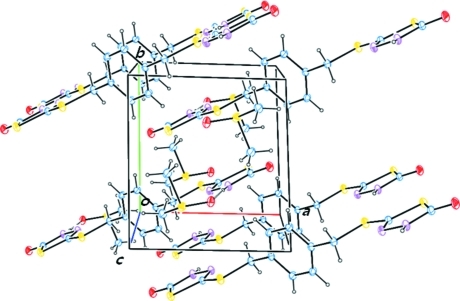
Part of the crystal structure of the title compound, showing molecules linked by intermolecular N—H···O hydrogen bonds (dashed lines).

## supplementary crystallographic information

### Comment

Polydentate macrocyclic compounds containing heterocyclic rings as subunits possess a variety of interesting properties. Heterocyclic units contain oxygen, nitrogen or sulfur, which provide the coordination sites allowing the heterocycles to form complexes with metals and act as effective hosts for different kinds of molecules (Dietrich *et al.*, 1993; Vogle, 1991). We studied on macrocyclic compounds composed of two 5-mercapto-2,3-dihydro-1,3,4-thiadizol-2-ones and two *p*-xylenes (Cho *et al.*, 1998, 1999, 2001). The NH of the title compound, α,α'-bis[(4,5-dihydro-5-oxo-1,3,4-thiazol-2-yl)thio]-*p*-xylene (I) is acidic enough to be alkylated in triethylamine with alkyl halide. The two NH functional groups can afford ring formation through an [2 + 2] alkylation.

The 5-oxo-1,3,4-thiadiazol-2-yl unit is planar, with an r.m.s. deviation of 0.004 Å from the corresponding squares plane defined by the seven constituent atoms. There is a crystallographic inversion center located in the middle of benzene ring. The bond distance of N4—C5 [1.281 (2) Å] is shorter than that of C2—N3 [1.337 (2) Å], which is consistent with double bond character. The thiadiazole and benzene rings are almost perpendicular to each other, with a dihedral angle 88.95 (6)°. The crystal structure is stabilized by the intermolecular N—H···O hydrogen bonds between the *p*-xylene compound and the dimethyl sulfoxide molecules (Fig. 1 and Table 1).

### Experimental

To a solution of α,α'-bis[(5-ethoxy-1,3,4-thiadiazol-2-yl)thio]-*p*-xylene (Cho *et al.*, 1999, 2001) (2.56 g, 6 mmol) in ethanol (20 ml), was added HBr (47%, 3.5 ml, 30 mmol), in one portion. The mixture was heated under reflux until the above *p*-xylene compound was disappeared on TLC. The solvent evaporated under reduced pressure to leave a solid residue, which was washed with water. The crude product was recrystallized from EtOH:THF = 3:1. Colorless crystals of (I) were obtained from its DMSO solution by slow evaporation of the solvent at room temperature. Yield 92%, m.p. 208–210°C; *R*_f_: 0.63 (n-hexane: EA = 5: 5); IR (KBr pellet, cm^-1^): 3120 (NH), 3062, 2950 (CH), 1656 (C═O), 1500, 1200; ^1^H NMR (DMSO-d_6_, p.p.m.): 12.95(2*H*, s, NH), 7.35(4*H*, s, C_6_H_4_), 4.32(4*H*, s, SCH_2_); ^13^C NMR (DMSO-d_6_, p.p.m.): 171.4 (C=O), 147.8 (C—S), 135.9, 129.2, (C_6_H_4_), 36.2 (SCH_2_).

### Refinement

Atom H3 of the NH group was located in a difference Fourier map and refined freely [refined distance: N—H = 0.91 (2) Å]. Other H atoms were positioned geometrically and refined using a riding model, with C—H = 0.93–0.97 Å, and with *U*_iso_(H) = 1.2*U*_eq_(carrier C) for aromatic and methylene, and 1.5*U*_eq_(carrier C) for methyl H atoms. DMSO molecule is disordered with an occupancy ratio of 0.879 (1):0.121 (1). For the minor component of the disordered DMSO molecule, bond length restraints of S═O = 1.49 (2) Å and S—C = 1.80 (2) Å were employed.

### Crystal data

**Table:**

C_12_H_10_N_4_O_2_S_4_·2C_2_H_6_OS	*Z* = 1
*M**_r_* = 526.74	*F*(000) = 274
Triclinic, *P*1	*D*_x_ = 1.434 Mg m^−^^3^
Hall symbol: -P 1	Mo *K*α radiation, λ = 0.71073 Å
*a* = 7.5723 (15) Å	Cell parameters from 5678 reflections
*b* = 8.3258 (17) Å	θ = 2.6–25.2°
*c* = 10.346 (2) Å	µ = 0.59 mm^−^^1^
α = 109.70 (4)°	*T* = 296 K
β = 95.74 (3)°	Block, colourless
γ = 91.15 (3)°	0.18 × 0.17 × 0.12 mm
*V* = 610.0 (2) Å^3^	

### Data collection

**Table:**

Bruker APEXII CCD diffractometer	2124 reflections with *I* > 2σ(*I*)
Graphite monochromator	*R*_int_ = 0.083
φ and ω scans	θ_max_ = 28.3°, θ_min_ = 2.1°
Absorption correction: multi-scan (*SADABS*; Bruker, 2002)	*h* = −10→10
*T*_min_ = 0.895, *T*_max_ = 0.923	*k* = −11→11
21342 measured reflections	*l* = −13→13
3039 independent reflections	

### Refinement

**Table:**

Refinement on *F*^2^	Primary atom site location: structure-invariant direct methods
Least-squares matrix: full	Secondary atom site location: difference Fourier map
*R*[*F*^2^ > 2σ(*F*^2^)] = 0.034	Hydrogen site location: inferred from neighbouring sites
*wR*(*F*^2^) = 0.100	H atoms treated by a mixture of independent and constrained refinement
*S* = 1.02	*w* = 1/[σ^2^(*F*_o_^2^) + (0.0506*P*)^2^] where *P* = (*F*_o_^2^ + 2*F*_c_^2^)/3
3039 reflections	(Δ/σ)_max_ < 0.001
162 parameters	Δρ_max_ = 0.16 e Å^−^^3^
3 restraints	Δρ_min_ = −0.22 e Å^−^^3^

### Special details

**Table:**

Geometry. All s.u.'s (except the s.u. in the dihedral angle between two l.s. planes) are estimated using the full covariance matrix. The cell s.u.'s are taken into account individually in the estimation of s.u.'s in distances, angles and torsion angles; correlations between s.u.'s in cell parameters are only used when they are defined by crystal symmetry. An approximate (isotropic) treatment of cell s.u.'s is used for estimating s.u.'s involving l.s. planes.
Refinement. Refinement of *F*^2^ against ALL reflections. The weighted *R*-factor *wR* and goodness of fit *S* are based on *F*^2^, conventional *R*-factors *R* are based on *F*, with *F* set to zero for negative *F*^2^. The threshold expression of *F*^2^ > 2σ(*F*^2^) is used only for calculating *R*-factors(gt) *etc*. and is not relevant to the choice of reflections for refinement. *R*-factors based on *F*^2^ are statistically about twice as large as those based on *F*, and *R*-factors based on ALL data will be even larger.

### Fractional atomic coordinates and isotropic or equivalent isotropic displacement parameters (Å^2^)

**Table:**

	*x*	*y*	*z*	*U*_iso_*/*U*_eq_	Occ. (<1)
S1	0.76776 (6)	0.37928 (6)	0.42321 (5)	0.07516 (18)	
C2	0.7758 (2)	0.3744 (2)	0.59474 (19)	0.0668 (4)	
N3	0.62432 (19)	0.2937 (2)	0.60130 (16)	0.0688 (4)	
H3	0.589 (3)	0.282 (3)	0.679 (2)	0.090 (7)*	
N4	0.50062 (17)	0.23800 (17)	0.48623 (14)	0.0611 (3)	
C5	0.55873 (19)	0.27358 (19)	0.38640 (17)	0.0549 (4)	
O6	0.89774 (16)	0.43193 (18)	0.68683 (15)	0.0946 (5)	
S7	0.44211 (6)	0.22149 (6)	0.22259 (5)	0.06970 (16)	
C8	0.2421 (2)	0.1210 (2)	0.25284 (16)	0.0598 (4)	
H8A	0.2729	0.0257	0.283	0.072*	
H8B	0.1848	0.2027	0.3249	0.072*	
C9	0.11735 (19)	0.05870 (19)	0.12143 (15)	0.0516 (3)	
C10	−0.0134 (2)	0.1597 (2)	0.09349 (16)	0.0610 (4)	
H10	−0.0228	0.2687	0.1563	0.073*	
C11	0.1302 (2)	−0.1018 (2)	0.02588 (16)	0.0603 (4)	
H11	0.2181	−0.1713	0.0421	0.072*	
S12	0.32235 (7)	0.21416 (7)	0.85488 (5)	0.0693 (2)	0.8786 (14)
O13	0.5091 (2)	0.2414 (3)	0.8277 (2)	0.0771 (6)	0.8786 (14)
C14	0.1951 (5)	0.1372 (4)	0.6870 (4)	0.0807 (9)	0.8786 (14)
H14A	0.2232	0.0218	0.639	0.121*	0.8786 (14)
H14B	0.2238	0.2084	0.635	0.121*	0.8786 (14)
H14C	0.0706	0.1402	0.6975	0.121*	0.8786 (14)
C15	0.2328 (6)	0.4158 (6)	0.9137 (4)	0.0964 (12)	0.8786 (14)
H15A	0.2907	0.4803	1.0039	0.145*	0.8786 (14)
H15B	0.1078	0.4017	0.9183	0.145*	0.8786 (14)
H15C	0.2511	0.4757	0.8509	0.145*	0.8786 (14)
S12A	0.3220 (5)	0.3284 (5)	0.7826 (4)	0.0717 (14)	0.1214 (14)
O13A	0.5108 (17)	0.3036 (17)	0.8234 (16)	0.057 (4)*	0.1214 (14)
C14A	0.209 (5)	0.126 (3)	0.729 (3)	0.093 (11)*	0.1214 (14)
H14D	0.29	0.0394	0.6939	0.14*	0.1214 (14)
H14E	0.1146	0.1194	0.6573	0.14*	0.1214 (14)
H14F	0.1595	0.1102	0.8058	0.14*	0.1214 (14)
C15A	0.250 (5)	0.410 (5)	0.952 (2)	0.087 (10)*	0.1214 (14)
H15D	0.3501	0.4642	1.0183	0.13*	0.1214 (14)
H15E	0.1998	0.3176	0.9746	0.13*	0.1214 (14)
H15F	0.1623	0.4918	0.953	0.13*	0.1214 (14)

### Atomic displacement parameters (Å^2^)

**Table:**

	*U*^11^	*U*^22^	*U*^33^	*U*^12^	*U*^13^	*U*^23^
S1	0.0514 (3)	0.0787 (3)	0.0898 (4)	−0.0150 (2)	−0.0004 (2)	0.0249 (2)
C2	0.0489 (9)	0.0614 (10)	0.0737 (11)	−0.0010 (7)	−0.0108 (8)	0.0069 (8)
N3	0.0541 (8)	0.0853 (10)	0.0560 (8)	−0.0119 (7)	−0.0137 (7)	0.0162 (7)
N4	0.0495 (7)	0.0731 (9)	0.0527 (7)	−0.0090 (6)	−0.0092 (6)	0.0159 (6)
C5	0.0436 (8)	0.0553 (8)	0.0607 (9)	−0.0018 (6)	−0.0025 (7)	0.0156 (7)
O6	0.0600 (8)	0.0931 (10)	0.0981 (10)	−0.0088 (7)	−0.0304 (7)	0.0017 (8)
S7	0.0588 (3)	0.0926 (3)	0.0573 (3)	−0.0129 (2)	−0.00617 (19)	0.0296 (2)
C8	0.0500 (9)	0.0749 (10)	0.0494 (8)	−0.0074 (7)	−0.0062 (7)	0.0184 (7)
C9	0.0442 (8)	0.0595 (9)	0.0460 (8)	−0.0028 (6)	−0.0026 (6)	0.0139 (7)
C10	0.0565 (9)	0.0579 (9)	0.0562 (9)	0.0060 (7)	−0.0038 (7)	0.0062 (7)
C11	0.0512 (9)	0.0627 (10)	0.0600 (9)	0.0109 (7)	−0.0066 (7)	0.0149 (7)
S12	0.0666 (3)	0.0796 (4)	0.0724 (4)	0.0135 (2)	0.0099 (2)	0.0388 (3)
O13	0.0570 (9)	0.1063 (17)	0.0751 (10)	0.0135 (10)	0.0017 (7)	0.0413 (12)
C14	0.0665 (16)	0.0774 (18)	0.085 (2)	−0.0001 (11)	−0.0046 (15)	0.0146 (15)
C15	0.0810 (19)	0.098 (2)	0.088 (2)	0.0245 (14)	−0.0073 (19)	0.007 (2)
S12A	0.064 (2)	0.092 (3)	0.070 (2)	0.0013 (18)	−0.0017 (17)	0.045 (2)

### Geometric parameters (Å, º)

**Table:**

S1—C5	1.7385 (16)	S12—O13	1.4964 (19)
S1—C2	1.784 (2)	S12—C15	1.757 (4)
C2—O6	1.220 (2)	S12—C14	1.802 (4)
C2—N3	1.337 (2)	C14—H14A	0.96
N3—N4	1.3772 (18)	C14—H14B	0.96
N3—H3	0.91 (2)	C14—H14C	0.96
N4—C5	1.281 (2)	C15—H15A	0.96
C5—S7	1.7406 (17)	C15—H15B	0.96
S7—C8	1.8202 (17)	C15—H15C	0.96
C8—C9	1.502 (2)	S12A—O13A	1.488 (13)
C8—H8A	0.97	S12A—C14A	1.758 (18)
C8—H8B	0.97	S12A—C15A	1.796 (18)
C9—C11	1.380 (2)	C14A—H14D	0.96
C9—C10	1.382 (2)	C14A—H14E	0.96
C10—C11^i^	1.379 (2)	C14A—H14F	0.96
C10—H10	0.93	C15A—H15D	0.96
C11—C10^i^	1.379 (2)	C15A—H15E	0.96
C11—H11	0.93	C15A—H15F	0.96
			
C5—S1—C2	88.75 (8)	C15—S12—C14	97.27 (19)
O6—C2—N3	127.16 (19)	S12—C14—H14A	109.5
O6—C2—S1	126.18 (16)	S12—C14—H14B	109.5
N3—C2—S1	106.66 (12)	H14A—C14—H14B	109.5
C2—N3—N4	119.02 (16)	S12—C14—H14C	109.5
C2—N3—H3	125.3 (14)	H14A—C14—H14C	109.5
N4—N3—H3	115.3 (14)	H14B—C14—H14C	109.5
C5—N4—N3	109.97 (14)	S12—C15—H15A	109.5
N4—C5—S1	115.59 (12)	S12—C15—H15B	109.5
N4—C5—S7	123.92 (12)	H15A—C15—H15B	109.5
S1—C5—S7	120.48 (10)	S12—C15—H15C	109.5
C5—S7—C8	98.72 (8)	H15A—C15—H15C	109.5
C9—C8—S7	109.26 (12)	H15B—C15—H15C	109.5
C9—C8—H8A	109.8	O13A—S12A—C14A	106.7 (13)
S7—C8—H8A	109.8	O13A—S12A—C15A	98.7 (13)
C9—C8—H8B	109.8	C14A—S12A—C15A	97.6 (18)
S7—C8—H8B	109.8	S12A—C14A—H14D	109.5
H8A—C8—H8B	108.3	S12A—C14A—H14E	109.5
C11—C9—C10	118.39 (13)	H14D—C14A—H14E	109.5
C11—C9—C8	120.59 (14)	S12A—C14A—H14F	109.5
C10—C9—C8	121.02 (14)	H14D—C14A—H14F	109.5
C11^i^—C10—C9	121.18 (14)	H14E—C14A—H14F	109.5
C11^i^—C10—H10	119.4	S12A—C15A—H15D	109.5
C9—C10—H10	119.4	S12A—C15A—H15E	109.5
C10^i^—C11—C9	120.43 (14)	H15D—C15A—H15E	109.5
C10^i^—C11—H11	119.8	S12A—C15A—H15F	109.5
C9—C11—H11	119.8	H15D—C15A—H15F	109.5
O13—S12—C15	107.4 (2)	H15E—C15A—H15F	109.5
O13—S12—C14	105.18 (15)		

Symmetry code: (i) −*x*, −*y*, −*z*.

### Hydrogen-bond geometry (Å, º)

**Table:**

*D*—H···*A*	*D*—H	H···*A*	*D*···*A*	*D*—H···*A*
N3—H3···O13	0.91 (2)	1.83 (2)	2.742 (3)	175.6 (19)

## References

[bb1] Bruker (2002). *SADABS*, *SAINT* and *SMART* Bruker AXS Inc., Madison, Wisconsin, USA.

[bb2] Cho, N. S., Hong, S. I., Park, Y. S. & Suh, I. H. (2001). *Bull. Korean Chem. Soc.* **22**, 1280–1282.

[bb3] Cho, N. S., Park, C. K., Kim, H. S., Choi, E. S. & Kang, S. K. (1998). *Bull. Korean Chem. Soc.* **19**, 103–106.

[bb4] Cho, N. S., Park, C. K., Kim, H. S., Oh, J. G., Suh, I. H. & Oh, M. R. (1999). *Heterocycles*, **51**, 2739–2746.

[bb5] Dietrich, B., Viout, P. & Lehn, J. M. (1993). In *Macrocyclic Chemistry: Aspects of Organic and Inorganic Supramolecular Chemistry* Weinheim: VCH.

[bb6] Farrugia, L. J. (1997). *J. Appl. Cryst.* **30**, 565.

[bb7] Farrugia, L. J. (1999). *J. Appl. Cryst.* **32**, 837–838.

[bb8] Sheldrick, G. M. (2008). *Acta Cryst.* A**64**, 112–122. 10.1107/S0108767307043930 18156677

[bb9] Vogle, F. (1991). In *Supramolecular Chemistry* Chichester: Wiley.

